# Two Novel Iflaviruses Discovered in Bat Samples in Washington State

**DOI:** 10.3390/v14050994

**Published:** 2022-05-07

**Authors:** Kate B. Juergens, John Huckabee, Alexander L. Greninger

**Affiliations:** 1 Department of Laboratory Medicine and Pathology, University of Washington Medical Center, Seattle, WA 98195, USA; katej16@uw.edu; 2 Vaccine and Infectious Disease Division, Fred Hutchinson Cancer Research Center, Seattle, WA 98109, USA; 3 PAWS (Progressive Animal Welfare Society) Wildlife Center, Lynnwood, WA 98087, USA; wilddoc@gmail.com

**Keywords:** iflavirus, RNA mNGS, bat metagenome, *Iflaviridae*

## Abstract

Arthropods are integral to ecosystem equilibrium, serving as both a food source for insectivores and supporting plant reproduction. Members of the *Iflaviridae* family in the order *Picornavirales* are frequently found in RNA sequenced from arthropods, who serve as their hosts. Here we implement a metagenomic deep sequencing approach followed by rapid amplification of cDNA ends (RACE) on viral RNA isolated from wild and captured bat guano in Washington State at two separate time points. From these samples we report the complete genomes of two novel viruses in the family *Iflaviridae*. The first virus, which we call King virus, is 46% identical by nucleotide to the lethal honeybee virus, deformed wing virus, while the second virus which we call Rolda virus, shares 39% nucleotide identity to deformed wing virus. King and Rolda virus genomes are 10,183 and 8934 nucleotides in length, respectively. Given these iflaviruses were detected in guano from captive bats whose sole food source was the *Tenebrio spp.* mealworm, we anticipate this invertebrate may be a likely host. Using the NCBI Sequence Read Archive, we found that these two viruses are located in six continents and have been isolated from a variety of arthropod and mammalian specimens.

## 1. Introduction

Native insectivorous bats (order *Chiroptera*) regularly feed on arthropods, giving direct entry of any pathogen within these bugs to one of the largest known mammalian reservoirs of zoonotic disease [1,2,3]. Through their distinct environmental biology and pathogen tolerant immune response, bats maintain an impressive virome naturally consisting of many viruses that reside within arthropod hosts [4,5]. Despite their common association as carriers of human disease, the virome of insectivorous bats largely consists of viruses derived from insects, and among this group is the family of viruses *Iflaviridae* [6].

Iflaviruses are non-enveloped, positive-sense single stranded RNA viruses with an average genome size of 9–11 kilobases in the family of *Iflaviridae* and order *Picornavirales* [7]. The *Iflaviridae* family is characterized as having a 5′ UTR internal ribosome entry site (IRES), a leader-protein virulence factor crucial for viral replication, and a 3C-like protease for processing of viral capsid proteins VP3, VP2, VP1, and VP4 (variably present) [8,9,10,11]. These picorna-like viruses are known to ubiquitously infect arthropods around the world and inflict varying degrees of disease within their hosts; while some are lethal, others are entirely benign [7]. Today, it is known that iflaviruses pose a threat to honeybee survival, have potential for acting as pest control, exhibit an extensive host range, and infect several invertebrate arthropod hosts known to be vectors of human disease [12,13,14,15].

Iflaviruses play an intriguing ecological role as both parasitic and commensal symbionts, and are recovered from a wide range of arthropods. Typically, this viral family is isolated from whole arthropod homogenates or environmental samples containing arthropod tissues. Recently, novel iflaviruses have been found in glandular tissues of the pink bollworm moth (a common pest of cotton fields), in whole leafhoppers that reside in rice crops, and in the midgut tissue of the Vietnamese walking stick bug [10,16,17]. The host of iflaviruses however may not be limited to only land-roaming arthropods. In 2015, a novel virus isolated from a tomato plant was found to be most closely related to the iflavirus family despite alignment to known picorna-like plant viruses [18]. Additionally, the chequa iflavirus is known to replicate in crayfish, a water-living crustacean [19].

The majority of these iflaviruses are introduced to bats and other mammals through the consumption of infected insects. Vector born exposure however is also possible, as iflaviruses infect arthropods such as the *Ixoodes holocyclus* and *Ixodes scapulares* ticks [20,21]. The broad mammalian host range of infected blood feeding insects in addition to the vast arthropod consumption by insectivores permits ample opportunity for iflavirus spillover events between insects and mammals. However, to date, replication of iflaviruses inside mammalian cells has not been demonstrated as possible.

These novel iflaviruses have largely been found through shotgun or transcriptomic sequencing. RNA metagenomic next generation sequencing (mNGS) returns rich data about the RNA virome of any given environmental or clinical specimen through random shotgun sequencing of RNA within a given sample. Increasing numbers of viral sequences are now available in the Sequence Read Archive (SRA), and a simultaneous emergence of cloud-based platforms that have made these data available to the scientific community at the petabase scale [22,23,24]. Parsing SRA files through these platforms provides raw reads containing viral sequences for researching a given genome of interest. Additionally, SRA data provide insight on the global distribution and host range of a given virus. This information is a quick and accessible resource for largely understudied viruses such as members of *Iflaviridae*, which we demonstrate in this study.

In this paper we performed mNGS on RNA extracted from bat guano in Washington State and discovered the full genomes of two novel iflaviruses. Complete genomes were resolved through rapid amplification of cDNA ends (RACE). Through data mining of the SRA, we extracted relevant information about their descriptive epidemiology and concluded that our viruses are distributed worldwide and have been isolated from a diverse set of candidate hosts.

## 2. Materials and Methods

### 2.1. Guano Sample Preparation and RNA Extraction for Deep Sequencing

For the 2016 samples, two sets of guano samples from putative *Myotis yumanensis* were collected from PAWS (Progressive Animal Welfare Society) and frozen at −20 °C (Lynnwood, WA, USA). For the 2021 samples, two fresh guano samples were collected over the course of three days from rescued bats at Happy Valley Bats in Bothell, Washington, and frozen immediately at −80 °C. All rescued bats were housed in the same shed and sequestered in nests with members of their own genus. While the bats were rescues and some sustained physical injury, to our knowledge there was no evidence of viral infection or illness. Based on anatomical study, the presumed species of each of the bats from which the guano samples originated were *Myotis californicus* and *Eptesicus fuscus*. After thawing guano at room temperature, a 1:10 dilution of each sample from each species was made by approximating one mg of the sample and submerging it in 1 mL of Roche STAR buffer. Prior to RNA extraction, samples were bead beat until completely homogenous. After bead beating, samples were spun down at 10,000 g for 60 s to pellet guano particles and 100 µL of supernatant was removed for RNA extraction. The *E. fuscus* sample was filtered with a 0.45 µM filter prior to extraction. Qiagen RNeasy kit was used for RNA extraction following the designated protocol with elution in 30 µL of molecular grade water [25].

### 2.2. Library Preparation and Deep Sequencing

Prior to cDNA synthesis, 18 µL of RNA was DNase treated using Invitrogen Turbo DNase following the recommended protocol with incubation at 37 °C for 30 min [26]. cDNA was synthesized through priming the first and second strands with 50 µM random hexamers. First strand cDNA was made following SuperScript IV (SSIV) Reverse Transcriptase (RT) kit with the RT reaction at the following temperatures: 23 °C for 10 min, 50 °C for 15 min, 10 °C for five min, and four °C for two min [27]. The second strand of cDNA was synthesized following Invitrogen Sequenase Polymerase kit with thermocycling at the following temperatures: 10 °C for 2 min, ramping up to 37 °C over the course of 8 min, and 37 °C for 8 min [28]. Libraries of each cDNA sample were prepared with Nextera XT (Illumina) kit with thermocycling for one cycle at 72 °C for 3 min and 95 °C for 30 s followed by 20 cycles of 95 °C for one min, 55 °C for 30 s, and 72 °C for 30 s with a final extension at 72 °C for 5 min [29]. Samples were purified with Ampure beads at 0.8× concentration and sequenced 1 × 100 bp on an Illumina NextSeq. Biosamples containing the King and Rolda virus reads taken from 2021 and 2016 have been deposited to the SRA (Bioproject PRJNA812346).

### 2.3. Metagenomic Analysis

Reads were uploaded to Chan Zuckerberg ID (CZ ID) Metagenomic Sequencing Pipeline (version 6.8, CZI, San Francisco, California, USA) with bats selected as host [30]. CZ ID interface is publicly available and an opensource pipeline has been published on GitHub [31]. A metagenomic heat map of all experimental conditions is available in the Supplementary Materials (Table S2). Confirmation of CZ ID metagenomic hits through directed alignment was performed using Geneious Prime Build 2021-07-19 12:20 Java Version 11.0.11+9 (64 bit) visualization software using Geneious Prime internal mapping algorithm at low sensitivity without iteration [32]. These reads were adapter and quality trimmed using Trimmomatic version 0.39 (-phred33 -threads 8 ILLUMINACLIP:adapters.fa:2:30:10 LEADING:3 TRAILING:3 SLIDINGWINDOW:4:15 MINLEN:75) [33]. Trimmed reads were then de novo assembled using single end SPAdes v3.15.3 generic assembly mode [34]. Alignments were carried out using MAFFT with 200 PAM scoring matrix, 1.53 gap open penalty and 0.123 offset value.

### 2.4. Phylogenetic Tree Generation

NCBI RefSeq *Iflaviridae* sequences available as of 10 February 2022 were selected for alignment using poliovirus reference sequence (NC_002058) as an outgroup [35]. The open reading frame (ORF) of each sequence was translated according to the designated annotation, and all *Iflaviridae* ORFs were aligned in Geneious Prime through MAFFT with Blosum 30 scoring matrix, a gap penalty of 1.53, and offset value of 0.123. The RdRp sequences were then selected within this alignment based on the RdRp genome annotation from NC_027917 after validating the annotation as accurate using HHpred [36]. Extracted *Iflaviridae* RdRp sequences were then again aligned with the annotated Poliovirus (NC_002058) RdRp. The Newick tree was made using a maximum likelihood distance method with IqTree multicore version 2.0.3 for Mac OS X (-m MFP -B 1000 -T AUTO) [37]. Each tree leaf was annotated with a silhouette of the putative host for the virus using BioSample metadata or literature review. Branches containing *Iflaviridae* isolated from likely mixed samples such as bat guano were left unlabeled.

### 2.5. Data Extraction of SRA for Descriptive Epidemiology

BigQuery search engine version 2.34.2 was used for metadata extraction based on SRA files with reads indexed as containing King and Rolda viruses [24]. Queries parsed the SRA based on the King and Rolda NCBI taxonomic IDs through the following command:

1. SELECT m.acc, m.sample_acc, m.biosample, m.sra_study, m.bioproject, tax.total_count
2. FROM ‘nih-sra-datastore.sra.metadata‘ as m, ‘nih-sra-datastore.sra_tax_analysis_tool.tax_analysis‘ as tax
3. WHERE m.acc = tax.acc and tax_id = 1911104

The BioSample accessions generated from this output were then put into Entrez Direct E-utilities efetch version 16.2 for data extraction of host and read counts for the calculation of RPM for King and Rolda virus in each sample [38]. Metadata generated from BigQuery and Entrez-utilities efetch search for King and Rolda viruses are available in the Supplementary Materials (Tables S3 and S4).

### 2.6. Genome Completion through RACE and RT-PCR

To complete King and Rolda virus genomes, 5′ and 3′ rapid amplification of cDNA ends (RACE) system was implemented on the original extracted viral RNA samples we described above. All primers designed for this experiment are available in the Supplementary Materials (Table S1). For 3′ RACE, first strand viral cDNA was synthesized using SSIV RT as described above with the oligo dT anchor primer added to a final concentration of 2 µM. After synthesis of the first strand, residual RNA was removed by adding 1 µL of RNAse H (ThermoFisher Scientific, Waltham, MA, USA) to the reaction and incubating at 37 °C for 20 min. PCR amplification of the 3′ end was performed using Takarabio CloneAmp PCR master mix with thermocycling at 98 °C for 2 min, and 35 cycles of 98 °C for 10 s, 57 °C for 15 s, 72 °C for 30 s, and a final extension step at 72 °C for 5 min [39]. The 3′ RACE viral specific primer and anchor primer were added to a final concentration of 0.4 µM for PCR amplification. Amplicons from both 3′ RACE products were run on a 1.2% agarose gel and extracted using Promega gel wizard kit [40]. The gel-extracted and purified amplicons were submitted for Sanger sequencing, and King and Rolda virus Sanger trace files are available in the Supplementary Materials (Files S1 and S2).

To obtain the 5′ end of each sequence, 5′ RACE using a template specific oligo was performed following the protocol from New England Biolabs (NEB): “5′ RACE with the NEB template switching RT Enzyme mix” [41]. All primers for this experiment are available in Supplementary Materials (Table S1). As 5′ RACE products did not yield a single clear amplicon, we created a next-generation sequencing library from those amplicons using the KAPA HyperPlus kit without performing the fragmentation step [42]. The King and Rolda virus 5′ RACE data from these libraries are available on the SRA (Bioproject PRJNA812346). The final 5′ sequence end for each virus was called by selecting the longest supported 5′ RACE amplicon. Complete King and Rolda virus sequences resulting from these RACE data have been deposited to GenBank (OM670244, OM670245).

### 2.7. RNA Folding

RNA structures were generated for the first 99 nucleotides (nt) in the 5′ untranslated region (UTR) and the full length 3′ UTR of each virus using RNAFold software version 2.5.0 [43]. The secondary structure was folded in puzzler format with the following options (-d2–noLP-t4). The optimal secondary structure output from this command at each nt with the corresponding sequence was input into Rosetta FARFAR2 as dot-bracket specified secondary structure using the generic score function [44]. An amount of 500 structures were generated in Rosetta and the top scoring output according to score/rms was used as the three dimensional model for analysis. PDBs of the King and Rolda virus 5′ and 3′ UTRs are available in the Supplementary Materials (Files S3–S6).

### 2.8. Genome Annotation with HHpred

After locating the longest ORF using an ATG start, segments of each translated ORF were queried in Hhpred and annotated according to the top hits. Smaller and smaller segments were interrogated in separate query submissions to annotate the remaining coding sequence to look for more distant alignments.

### 2.9. RT-qPCR of Rolda and King Virus in Mealworm and Bat Samples

PowerUp SYBR Green Master Mix was used following the manufacturer’s protocol to approximate copy numbers of King and Rolda virus per mg of bat guano and mealworm tissue [45]. Each bat guano sample was measured by subtracting the blank negative control Eppendorf tube from an identical Eppendorf tube containing the guano sample. For mealworms, the average mass of the *Tenebrio molitor* mealworm was used as an estimate of tissue mass (105 mg). A total of 10 whole mealworms were entirely submitted for RNA extraction in ten separate tubes. A negative control containing only Roche STAR buffer was extracted along with bat and mealworm samples to rule out contamination. Samples were bead beat until completely homogenized. A volume of 200 µL of completely homogenized guano and mealworm tissue supernatant was filtered through a 0.22 µM filter. The 200 µL filtrate was then extracted using Qiagen RNeasy protocol with an elution in 30 µL of water as described above. Single stranded cDNA was synthesized with SSIV RT as described above using a King_9223R or Rolda_8224R at a concentration of 0.2 µM. Internal qPCR standards were generated via amplification of leftover extracted bat guano RNA using Rolda 7685F-8224R and King 8812F-9524R. Amplicons were gel-purified using the Promega gel wizard kit and quantitated using the dsDNA HS kit on the Qubit 3.0 Fluorometer. Each standard amplicon was diluted down to 500,000 copies per µL (through calculation based on the concentration) and subject to a ten-fold serial dilution. qPCR was performed using the PowerUp SYBR Green Master Mix following kit specifications using quarter reactions. An amount each of 1 µL of the standards, STAR buffer negative control, and experimental input cDNA was aliquoted into each condition with three replicates of each condition. The reaction was run at 50 °C for 5 min, 95 °C for 2 min, followed by 40 cycles of 95 °C for 1 min, 54 °C for 15 s, and 60 °C for 1 min with a final melt curve analysis at 95 °C for 15 s, 54 °C for 1 min, and a final temperature increase to 95 °C at 0.15 °C/s. The King virus reactions were Sanger sequenced for confirmation due to the low viral copy number measured. The final Sanger trace is available in the Supplementary Materials.

## 3. Results

### 3.1. Metagenomic and qPCR Analysis of Bat Guano from Washington State in 2016 and 2021

We originally detected two novel iflaviruses while metagenomically sequencing nuclease-treated bat guano collected from two putative *Myotis yumanensis* from a local animal welfare society in July 2016. De novo assembly of 750,352 reads from specimen 16-2260 yielded 7550 nucleotide (nt) and 2897 nt contigs that further assembled to 10,193 nt contig containing a 9114 nt open reading frame (ORF) that most closely aligned at 32.7% by amino acid by blastp to deformed wing virus at the time. We named this sequence King virus strain UWV2, given the lack of definitive host species association, and deposited it in GenBank (NC_031749). Re-alignment of the 16-2260 sequencing reads to the contig revealed the likely presence of more than one strain of King virus in the specimen based on a number of mixed alleles present in the capsid region occurring at frequencies around 50%. De novo assembly of 234,984 reads from specimen 16-1811 yielded an 8593 nt contig containing an 8017 nt ORF truncated at the 3′ end that most closely aligned at 32.0% by amino acid by blastp to diaphorina citri picorna-like virus. We named this sequence Rolda virus strain UW1, again given the lack of definitive host species association, and deposited it in GenBank (KX779452). Direct alignment of 16-1811 sequencing reads to the contig also demonstrated the likely presence of more than one species of Rolda virus in the specimen based on the identification of numerous minor alleles. In addition, the second longest contig from the de novo assembly of specimen 16-1811 yielded a 6911 nt contig of King virus (strain UWV1, KX779453) that shared 89.6% nucleotide identity with King virus and we called this strain UWV2.

Five years later, we returned to metagenomically sequencing bat guano from Washington State. We extracted RNA from two bat guano samples collected from *Myotis californicus* and *Eptesicus fuscus* genuses from a local bat rescue operation and prepared mNGS sequencing libraries. We ran these samples through Chan Zuckerburg ID (CZ ID) which resulted in an average of 35.5 million non-host reads recovered for each of the samples after adapter/quality trimming and host subtraction. We observed between $1.47\times 10^{-2}\%\;\mathrm{and}\;1.63\times 10^{-1}\%$ for King virus and $2.19\times 10^{-3}\%\;\mathrm{and}\;1.35\times 10^{-1}\%\;$for Rolda virus of non-host reads taxonomically assigned. The *E. fuscus* specimen had the greatest amount of King virus at 1240 reads per million (RPM) on target covering 95.4% of the previously deposited genome by NCBI NT GSNAP alignment, while *M. californicus* specimen had the greatest amount of Rolda virus at 1730 RPM on target and coverage breadth of 100% by GSNAP alignment to NCBI NT.

We detected these viruses in bats held as captive rescues whose sole food source was the *Tenebrio molitor* mealworm. Despite members of the *Iflaviridae* family often being discovered in mammalian fecal matter, it has been established that the definitive host of these viruses is arthropods. We thus suspected that both viruses would be detectable in the mealworms being fed to the bats, and attempted quantification of King and Rolda viral copy numbers in the mealworm tissue. Ten whole live blended mealworms and the bat guano homogenate were analyzed through RT-qPCR which confirmed King and Rolda viruses at detectable levels within the mealworms. The King virus had low copy numbers within the mixed mealworms at an estimated 6.6 copies per mg of mealworm tissue yielding an average cycle threshold (Ct) of 38 per replicate. Due to a high Ct and low copy number, we confirmed the amplicon specificity via Sanger sequencing. In contrast, Rolda virus was detected at substantially higher levels within mealworm tissue at 1250 copies per mg. In the guano, King virus was detected in the *E. fuscus* sample at a level of 250 copies per mg of guano while Rolda virus had an estimated 38 viral copies per mg of guano. These data suggest that the King and Rolda viruses we isolated from the bats were likely ingested from actively infected *Tenebrio molitor* mealworms.

### 3.2. Genome Completion and Analysis of Novel King and Rolda Viruses

**Figure 1:**
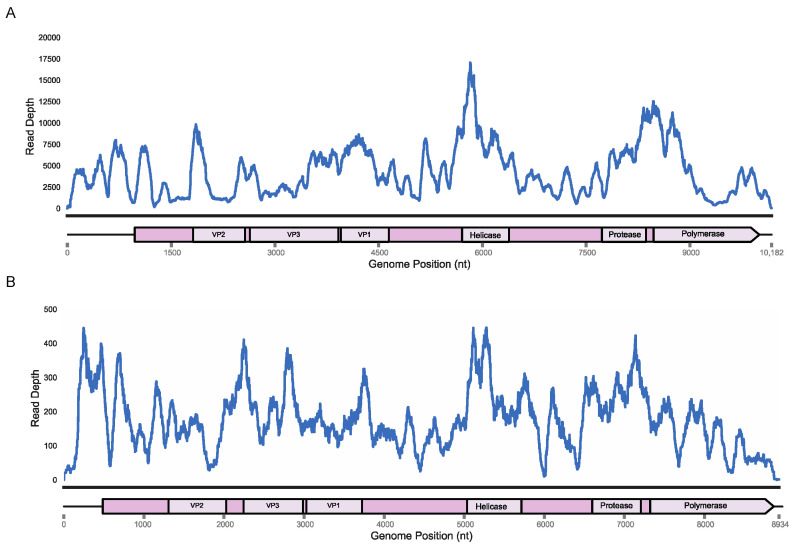
Read depth plotted according to nucleotide position for both King virus (**A**), and Rolda virus (**B**), genomes from original samples and annotations based on HHPred homology.

Based on the high viral loads of these specimens and de novo assembly contigs that covered >90% of the previously recovered genomes, we used these samples for 5′ and 3′ RACE in order to complete the viral genomes left incomplete in 2016. The 3′ RACE using a tailed oligo-dT primer recovered a 162 nt long 3′ UTR for King virus (OM670244) and a 60 nt long 3′ UTR for Rolda virus (OM670245) through Sanger sequencing of PCR amplicons. The 5′ RACE using a template switching oligo followed by deep sequencing of the gene-specific PCR amplicons resulted in 973 and 535 nt 5′ UTR regions in King and Rolda viruses based on distance to the ORF ATG start codon. Remapping of the original reads from *E. fuscus* and *M. californicus* samples to the 10,169 nt King virus and 8920 nt Rolda virus showed read coverage across nearly the entire span of the genome aside from a coverage drop off in the UTRs resolved through RACE (Figure 1). We named these new strains King virus isolate UWV3 and Rolda virus isolate UWV2. The 5′ RACE yielded two uracil nucleotides at the far 5′ end for the King virus, matching the starting nucleotides of the successful deformed wing virus reverse genetics system, in addition to that of the *Picornaviridae* family [46,47]. However, 5′ RACE of Rolda virus yielded two adenine nts at the far 5′ end, indicating that the viral genome may not yet be entirely recovered or is genetically distinct.

**Figure 2:**
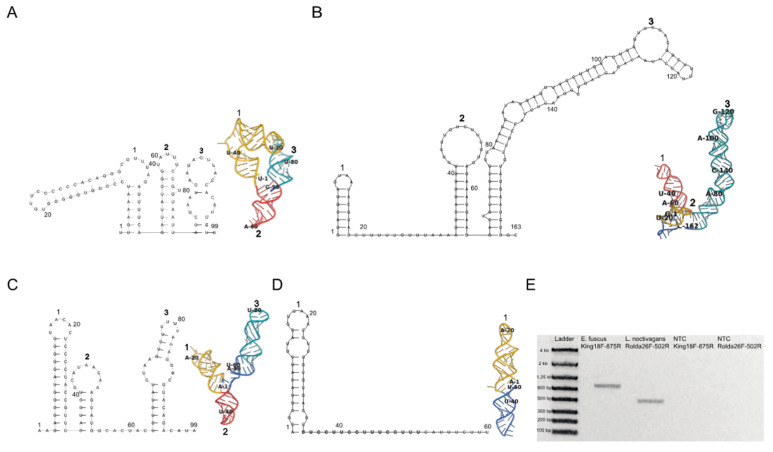
King virus 5′ and 3′ UTR (**A**,**B**), and Rolda virus 5′ and 3′ UTR (**C**,**D**). Folding was implemented with the first 99 nt of each virus’ 5′ UTR and the full length 3′ UTR. Two- (left) and three- (right) dimensional structures were folded using RNAFold and Rosetta FARFAR2. Corresponding hairpins are labeled between each 2D and 3D structure. The first hairpin in the three dimensional fold is colored yellow, followed by the second and third hairpins (if present) colored in red and teal. Nucleotides that are predicted to not partake in Watson–Crick base pairing for loop formation by RNAFold are colored in cobalt. Every 20 nucleotides are labeled in both 2D and 3D structures. (**E**) Gel depicting the 5′ UTR RT-PCR products and corresponding negative controls for confirmation of 5′ UTR sequence observed in 5′ RACE.

Completion of King and Rolda virus genomes allowed for investigation of structural elements within 5′ and 3′ UTRs (Figure 2). Since RNA structures located in *Picornavirales* non-coding regions are integral for genome replication, translation, and viral assembly [48], we evaluated potential structural RNA virulence factors present in our sequences through secondary structure RNA folding with RNAfold. King virus folded into three consecutive hairpins of similar height (~12 nt) in the 3′ UTR, and three structurally divergent hairpins in the 5′ UTR (Figure 2A,B). The third hairpin in the 3′ UTR was long relative to the other RNA loops spanning a length of 95 nt and had a unique fold while locating itself closer to the polyA sequence. Rolda virus also had three back-to-back hairpins in the 5′ UTR, and a single loop in the 3′ UTR which immediately followed the stop codon (Figure 2C,D). To represent the structural motifs as they likely exist in vivo and further interrogate support for the two-dimensional folds, we generated 500 three dimensional models via fragment assembly and full atom refinement sampling of these initial secondary structures using Rosetta FARFAR2. The highest scoring structures generated by Rosetta were in agreement with the two-dimensional folds containing analogous Watson–Crick base paring within the hairpins. Three-dimensional analysis also revealed that the second hairpin in both 5′ UTRs of each virus was inverted, likely due to atomic crowding and thus inversion of the loop for optimization of Lennard–Jones potential. While the initial hairpin maintained A1 as paired to U36 in the Rolda virus 3′ UTR hairpin, the remainder of the sequence folded around itself forming polar contacts with the remaining sequence according to Rosetta. To further validate these 5′ UTR sequences in addition to the deep sequencing 5′ RACE, we also performed RT-PCR from near the 5′ end (Figure 2E).

**Figure 3:**
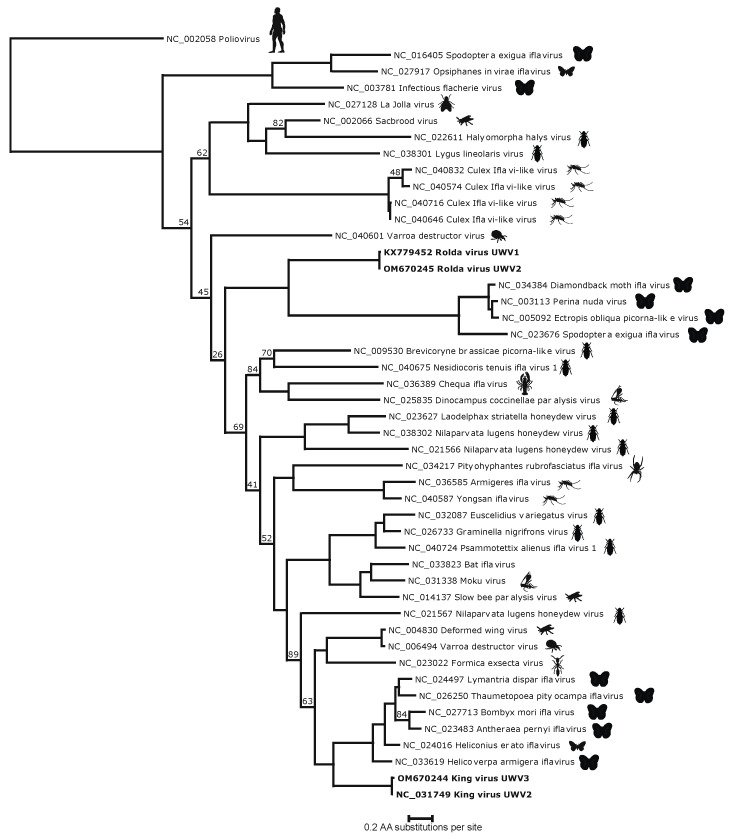
Phylogenetic tree generated by pairwise alignment of the translated RdRp of annotated non-redundant high-quality sequences. King and Rolda viruses are shown in bold. The putative host for each virus is shown to the right of the tree as a silhouette. Branch nodes with less than 95% are shown at respective nodes.

HHPred analysis of the King and Rolda virus ORFs yielded the canonical *Picornavirales* VP2, VP3, and VP1 capsid structures, in addition to 2C helicase, 3C protease, and 3D polymerase. Phylogenetic analysis of the conserved 3D polymerase compared with other *Iflaviridae* in NCBI RefSeq using poliovirus as an outgroup demonstrated that both King virus and Rolda virus clustered uniquely and separately with other moth iflaviruses (Figure 3). Within this conserved region, King virus shared 51.1% amino acid identity with Lymantria dispar iflavirus 1 and Rolda virus most closely aligned with 31.6% amino acid identity to Perina nuda virus. Pairwise alignment of high resolution (2.6 A) crystal structure sequences of slow bee paralysis virus VP1 (RCSB accession 5J98) showed 36.2% (King) and 22.1% (Rolda) amino acid identity to the VP1 sequences identified by HHPred. VP2, the first structural protein in both sequences was minimally 200 aa away from the putative start codon; consistent with space for a leader protein (L protein). However, this region in neither virus showed significant alignment with known PDB structures by HHPred. The 2A region of both King virus and Rolda virus was found to share distant homology by HHPred with the GCN1 protein, including a 92.5% probability for the Rolda virus 2A.

### 3.3. Geographic Distrubution and Data Availability of King and Rolda Viruses

**Figure 4:**
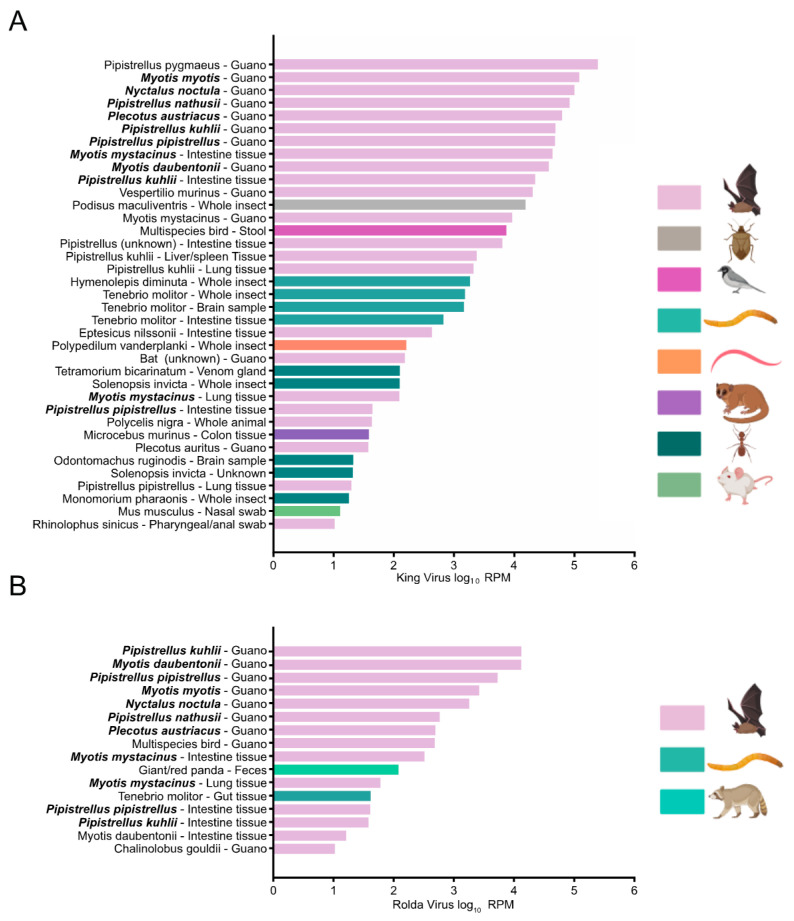
Bar chart depicting log_10_ RPM of non-redundant biosamples containing at least 10 RPM from BigQuery search of King (**A**) and Rolda (**B**) virus taxons. Samples containing both King and Rolda viruses are shown in bold.

Because of our initial deposition of King and Rolda virus into GenBank in 2016 and inclusion within RefSeq, each record in the Sequence Read Archive (SRA) has been indexed for both viruses. We took advantage of this fact to understand the descriptive epidemiology of King and Rolda virus by using BigQuery to comprehensively search the SRA for these viruses. In total, we found 37 SRA records with reads per million (RPM) >10 for King virus and 16 records for Rolda virus (Figure 4). Similar to our work, the majority of detections for King and Rolda virus occurred in bat guano. The median RPM per sample was 81.7 times higher for King virus than Rolda virus. Direct alignment of high RPM records to the complete genomes of King and Rolda virus confirmed our 2021 genomes described here. SRA files containing above 5000 RPM of Rolda virus were only observed in bat guano and tissue samples from Switzerland, where a large survey of bats had been undertaken [49]. King and Rolda virus were found in the same sample 11 times, each time from the same Swiss project. We observed that these viruses were not only present in bat guano, but also bat intestinal and lung tissue. King and Rolda virus were also found in a variety of arthropods, including mealworms, sleeping chironomids, fire ants, and snapping ants, suggesting a potentially broad host range around the world.

## 4. Discussion

We have reported and described the complete genomes of two novel iflaviruses identified in bat guano from Washington State. Phylogenetic placement of these viruses puts them as unique species within the *Iflaviridae* family, which, to date, lacks definitive genera. Additionally, we directly detected and quantified these viruses in a putative arthropod host, the *Tenebrio molitor* mealworm, and found additional potential hosts via mining of metagenomic and transcriptomic data in the Sequence Read Archive (SRA).

The early deposition of these viral sequences in 2016 allowed for rapid searching of SRA data that, to date, remains expensive and has been described sparingly for RNA viruses [22]. From this data we found that King and Rolda viruses have been recovered worldwide from a wide distribution of diverse arthropod hosts. A well-documented example of iflaviral dynamic host tropism is the horizontal transmission of sacbrood and deformed wing viruses between the Varroa destructor mite and *Apis Mellifera* honeybee—two phylogenetically distant arthropods [50,51]. These examples suggest that iflaviruses, and in particular King and Rolda viruses, make use of highly conserved receptors that allow them to be detected in such a diverse set of arthropod hosts. Indeed, to date, no entry receptor has been described for any iflavirus. Certainly, greater use of sequencing databases such as the SRA can help to rapidly determine potential host species of iflaviruses and aid in triangulating conservation of putative receptors.

In addition to confirming canonical picorna-like virus genes within these two iflaviruses, protein homology search through HHpred identified structures resembling the GCN1 translational control protein within the 2A region. We noted similar HHPred scores in the 2A region from other *Iflaviridae* species, including deformed wing virus and sacbrood virus. It has been established that *Picornaviridae* maintain proteinase genes in the 2A region (2A^Pro^) that cleave host translational initiation factors thus suppressing eukaryotic protein synthesis [52]. GCN1 is activated by ribosomal stalling and disome formation and functions to block translation through activation of GCN2 which phosphorylates eIF2alpha [53,54]. The area of homology detected in the viral 2A region includes the HEAT repeat domain of the eEF3-like region of GCN1 which contacts ribosomal protein S19 and GCN20. This raises the intriguing possibility that *Iflaviridae* 2A may hinder translational inhibition by blocking GCN1-GCN20 complex formation, thus preventing activation of GCN2 and downstream phosphorylation of eIF2alpha. This would imply *Iflaviridae* 2A region has coding sequence responsible for decreasing host protein synthesis through eIF2alpha. The e-values for these models however are by no means definitive, and alternatively the overall homology may largely be driven by the alpha helical repeats found in HEAT repeat containing proteins.

Finally, we found that folding of King and Rolda RNA in two and three dimensions resulted in identical hairpin motifs with analogous Watson–Crick base pairing between each 2D and 3D model in both 5′ and 3′ King and Rolda virus UTRs. These structures generated in silico provide insight to in vivo conformation of viral RNA in these regions which may have implicit function. Moving forward, greater use of 3D RNA structures in UTRs has the potential for revealing patterns of nucleic acid motifs that may serve as virulence factors.

Our study is chiefly limited by the lack of experimental and phenotypic data for these viruses. Certainly, more work is required to determine the definitive hosts for King and Rolda viruses, and confirmation of the completeness of these and other viral genomes relies on the reverse genetics system as performed for deformed wing virus. The 5′ end of King virus matched the canonical terminal uracil nucleotides described in *Picornaviridae* and deformed wing virus, strongly indicating that this genome is complete. More work may be required for completing the Rolda virus genome, as the 5′ end lacks this signature. Our data generated through the SRA, that offers insight into the host range for King and Rolda viruses, will also require direct experimental confirmation to determine if they are associated with any pathogenicity in these candidate arthropod hosts. As more King and Rolda viruses and other sparsely documented members of *Iflaviridae* are discovered through mNGS, SRA data will clarify the significance of these arthropods as definitive hosts. In the wake of the SARS-CoV-2 pandemic and expanded viral discovery efforts in bats and other mammalian species, we anticipate widespread detection of King and Rolda viruses and other members of *Iflaviridae* over the coming years [55].

## Data Availability

King and Rolda virus genomes are deposited at GenBank (Accessions OM670244 and OM670245). All SRA data can be found under Bioproject Accession PRJNA812346. All other data are presented in this manuscript or available in supplementary materials.

## Supplementary Materials

The following supporting information can be downloaded at https://www.mdpi.com/article/10.3390/v14050994/s1, File S1: king_3_RACE_trace_SF1.ab1; File S2: rolda_3_RACE_trace_SF2.ab1; File S3: king_5_utr.pdb; File S4: king_3_UTR.pdb; File S5: rolda_5_UTR.pdb; File S6: rolda_3_UTR.pdb; Table S1: ST1_Primers.csv; Table S2: ST2_IDSeq_Heatmap.csv; Table S3: ST3_King_BigQuery.csv; Table S4: ST4_Rolda_BigQuery.csv
